# Understanding contexts and mechanisms through which video based benchmarking promotes alignment of examiners’ scoring in objective structured clinical exams

**DOI:** 10.1007/s10459-025-10454-3

**Published:** 2025-07-04

**Authors:** Rebecca Jane Edwards, Peter Yeates, Janet Lefroy, Robert McKinley

**Affiliations:** 1 School of Medicine, University of Leicester, Leicester, UK; 2 School of Medicine, Keele University, Keele, UK

**Keywords:** Assessment, OSCEs, Faculty development, Rater cognition

## Abstract

Aligning examiners judgements to a shared standard is desirable within Objective Structured Clinical Exams (OSCEs) because it accords with OSCEs’ epistemic assumptions and purpose. Video-based benchmarking (VBB) involves examiners scoring station specific videos and comparing their judgements against an agreed score. Despite promising potential to enhance examiner alignment, VBB has been little researched. As the intervention’s efficacy is likely influenced by context, we used Realist Evaluation to study how, why and under what circumstances VBB may aid examiner alignment. Within stimulated interviews, examiners viewed videos of year-3 student OSCE performances and received expert-panel derived benchmark information. Different participants experienced adaptations of VBB, including varying benchmark performance standards, and different timings of receiving the benchmark information. Our resulting middle range theory suggests that despite conscientious preparation with written material, examiners’ expectations remain uncertain until encountering concrete performance examples. Judging a video serves this role, whilst comparison to a standard allows examiners to locate their judgement. Examiners experience dissonance when their scores are discrepant, and several contextual factors mediate whether they dismiss the suggested standard or adjust their perspectives. Examiners’ degree of engagement with video observation and score reflection is critical to VBB’s efficacy, and is influenced by examiner factors and other procedural contexts. These procedural contexts underpin tentative recommendations for practice which may enhance the likelihood of VBB’s effectiveness. When conscientious examiners engage effectively with well-constructed benchmarks, they perceive themselves as more prepared and aligned in their judgements because comparative reflection produces adjustment to their frame of reference.

**Supplementary Information:**

The online version contains supplementary material available at 10.1007/s10459-025-10454-3.

## Introduction

Differences in the judgements of assessors while judging the same performance can be considered to arise due to assessor “error” or due to legitimate differences in opinion between expert assessors (Gingerich et al., 2014). Whilst the latter approach is embraced within social constructivist approaches to assessment (Govaerts et al., 2007), where value is placed on differing perspectives, the ‘variability as error’ perspective predominates for summative objective structured clinical exams (OSCEs) (Newble, 2004) because it aligns with their typically comparatively objectivist purpose (Tavares et al., 2019). The OSCE’s role is generally to rank or measure candidates on a pre-defined range of comparable tasks under equivalent conditions often to determine progression or career opportunities (Norcini et al., 2018). In such settings, it is desirable to harmonise examiners’ judgements as far as possible.

A range of strategies have been used to harmonise examiners’ judgements. Foremost is the role of assessment design, acknowledging that adequate sampling of a blueprint with a sufficient number of stations will typically produce acceptable reliability as examiner differences tend to counter-balance across a sufficient sample (Eva, 2018; Swanson & van der Vleuten, 2013). At an examiner level, guiding examiners’ judgements with enhanced assessment rubrics (i.e. descriptions of what scores to allocate for what behaviours) has had limited success (Landy & Farr, 1980). Another recent approach has been to measure and adjust for examiner differences (Yeates et al., 2021). However, the major focus remains on assessor training.

Assessor training within health professionals education draws on principles established in occupational psychology such as: performance dimension training (ensuring examiners understand the scale which is employed); rater error training (training examiners about the typical errors which assessors may make), behavioural observation training (focusing on what behaviours should be observed) and frame of reference training (aiding examiners to develop a sense of the performance that should be awarded scores at each level of the rating scale, based on either written or video-based examples of performance, often combined with elements of the other 3 approaches) (Woehr & Huffcutt, 1994). Whilst a meta-analysis of studies investigating frame of reference training has shown a moderate effect (Cohen’s d = 0.5) (Roch et al., 2012), its effects in health professionals’ education are often more modest. Holmboe et al. (2004) found that training increased the severity of assessors scoring without substantially reducing the range of their scores. Cook et al. (2009) found no statistically significant increase in inter-rater reliability following a training workshop although the sample size produced comparatively broad confidence intervals. More recently, Kogan and colleagues (2023) achieved a moderate increase in assessor accuracy (58.1% vs. 49.7%) through training, but this involved six hours of initial training followed by 3 further asynchronous spaced learning modules over a 3-month period, an intensity which is not always practical.

A more focused form of frame of reference training is “examiner calibration” or “examiner benchmarking”. This typically involves examiners watching videos of candidates performing the *specific* task (or station) they are about to examine a relatively *short time* (for example, within hours or a few days) before the assessment. Examiners then reflect on their own impressions of the performance in relation to those of either their peers or an expert panel. This differs from other approaches which use generic training performances and may occur weeks or months prior to the exam. These factors are important as case specificity (the tendency for clinicians performance to vary across different topics) (Schauber et al., 2018), is likely to influence assessors’ judgements; and rater training, which is inherently a form of learning, may be expected to attenuate over time (Murre & Dros, 2015). Further, unlike the common pre-OSCE practice of examiners discussing their expectations of a station and deciding collectively how to attribute marks, which relies on abstract conceptualizations of students’ performance, examiner benchmarking uses concrete examples. This is important as training based on video has been shown to have a greater impact than training based on discussion of performance (Sisco & Leventhal, 2007).

Noting this, we define the object of our inquiry, “Video-based benchmarking” (VBB), as an intervention which aims to align examiners’ judgments within OSCEs through a process of judging station-specific video-based performance(s) and reflecting on information provided about the performance(s), within a short interval prior to the OSCE. Whilst this could be considered an adaptation of frame of reference training, its task-specificity and timing distinguish it from previous work on faculty development within medical education.

Literature on the effectiveness or operation of VBB from health professionals’ education is limited. Both Martin et al. (1998) and Hawkins et al. (2012) showed significant improvements in students’ self-assessment after respectively watching four benchmarking videos and just one benchmarking video. By contrast, Ward et al. (2003) found no effect on residents’ self-assessment with a similar intervention, although both groups of their participants (as well as being more clinically experienced than the previous studies) had watched a video of an ideal performance which could have contaminated the intervention. Crossingham et al. (2012) used 3 video-based benchmarks (poor, medium and good performances) to train anaesthetists in assessment of non-technical skills. They found no improvement in assessment accuracy, but their approach was less task-specific and had a longer interval between training and assessment than in our proposed definition.

At a theoretical level, assessors are understood to have what are known as “individual performance theories”, which describe their expectations of learners at different performance levels (Govaerts et al., 2013). The original intent of frame of reference training was to align individual performance theories with a prescribed frame of reference by comparison and exemplification (Woehr & Huffcutt, 1994). Assessors are understood to make judgments through a process of observation, processing, and integration. This involves considering what an assessor understands of the dimensions of performance, categorisation of observation, comparison with previous exemplars, content specificity of the judgement, inference, weighting and then making a judgement, and translating it into a scale (Gauthier et al., 2016). Assessors are well known to be influenced by recently observed performances (Shaw et al., 2021; Yeates et al., 2015). This suggests that VBB would offer examiners shared recent exemplars which are content-specific and clearly related to the relevant performance domains. As a result, it is reasonable to posit that it will have a beneficial influence on aligning examiners’ judgements.

Despite this, several contextual variables within these literatures appear to have the potential to influence the effectiveness of VBB. Firstly, the number of benchmarking videos varies between studies and it is unclear how many are required. Next, the level of the performances shown varies. Examiners experience greatest uncertainty about performances at the pass-fail borderline (Yeates, O’Neill, Mann, & W Eva, Yeates et al., 2013a, b), so these may offer the most meaningful benchmarks. Conversely, providing performances at the top and bottom of the scale accords with the original intention of frame of reference training (Uggerslev & Sulsky, 2008), but such performances have the potential to bias examiners’ judgements through either contrast effects, where assigned scores for a current performance shift away from the level of a preceding performance (Yeates et al., 2012; Yeates, O’Neill, Mann, & W Eva, Yeates et al., 2013a, b) or assimilation effects, where performance scores shift toward the level of the previously viewed performance (Murto et al., 2021; Shaw et al., 2021). It is unclear whether examiners should know the level of the performances they observe (e.g. low, borderline or high with the intention of cueing attention to relevant aspects) or whether examiners should score performances ‘blind’ to enable the best comparison of their existing frame of reference with that of ‘experts’. Frame of reference interventions vary between whether participants discuss their interpretations in groups or omit discussion (Kim et al., 2022). Experienced examiners are expected to have well-developed individual performance theories which reflect their own perspectives (Govaerts et al., 2013; Yeates et al., 2013a) whilst more novice examiners often experience greater uncertainty in judgement (Berendonk et al., 2013). Examiners’ varied clinical backgrounds will mean that they have different levels of familiarity with the station content. Consequently, whilst the cited literature and theory suggest that VBB is likely to be effective in at least some circumstances, it is unclear how VBB may work, and particularly how it may operate differently under different conditions or for different people, which may in turn influence whether or not it succeeds in its aim of aligning the judgements of OSCE examiners. Given the potential benefits of VBB to examiner standardisation within OSCEs, but the uncertainty over both its operation and effectiveness, we decided to study these issues by asking: In which contexts and by what mechanisms does VBB influence OSCE examiners’ expectations, perceptions, and judgements of candidate performance?

## Methods

### Theoretical stance

To address this question, we adopted Realist Evaluation as our methodological strategy. This asserts that whilst the phenomenon of study has mind-independent existence (ontological realism), our knowledge of it is socially and contextually structured and both fallible and transient (epistemological relativism) (Bhaskar, 2014). This philosophy holds that reality exists at three levels: the empirical (what humans experience or observe), the actual (the events which occur, whether or not they are observed) and the real (the causal mechanisms which are responsible for the events which occur) (Fletcher, 2017). Within this paradigm, the focus of knowledge building is on understanding mechanisms of causation, or *why* particular outcomes arise in particular circumstances by using what can be observed in the empirical, and sometimes actual, to deduce the real. This relies on judgemental rationality, or weighing the value of one theory over another, as a way to explain what was observed (Isaksen, 2022). Within Realist Evaluation, these causal theories are organised into constellations of “context”, “mechanism” and “outcome” with a focus on explaining how an intervention in a given context gives rise to particular mechanisms which lead to given outcomes (Pawson & Tilley, 1997).

### Initial programme theory

**Fig. 1:**
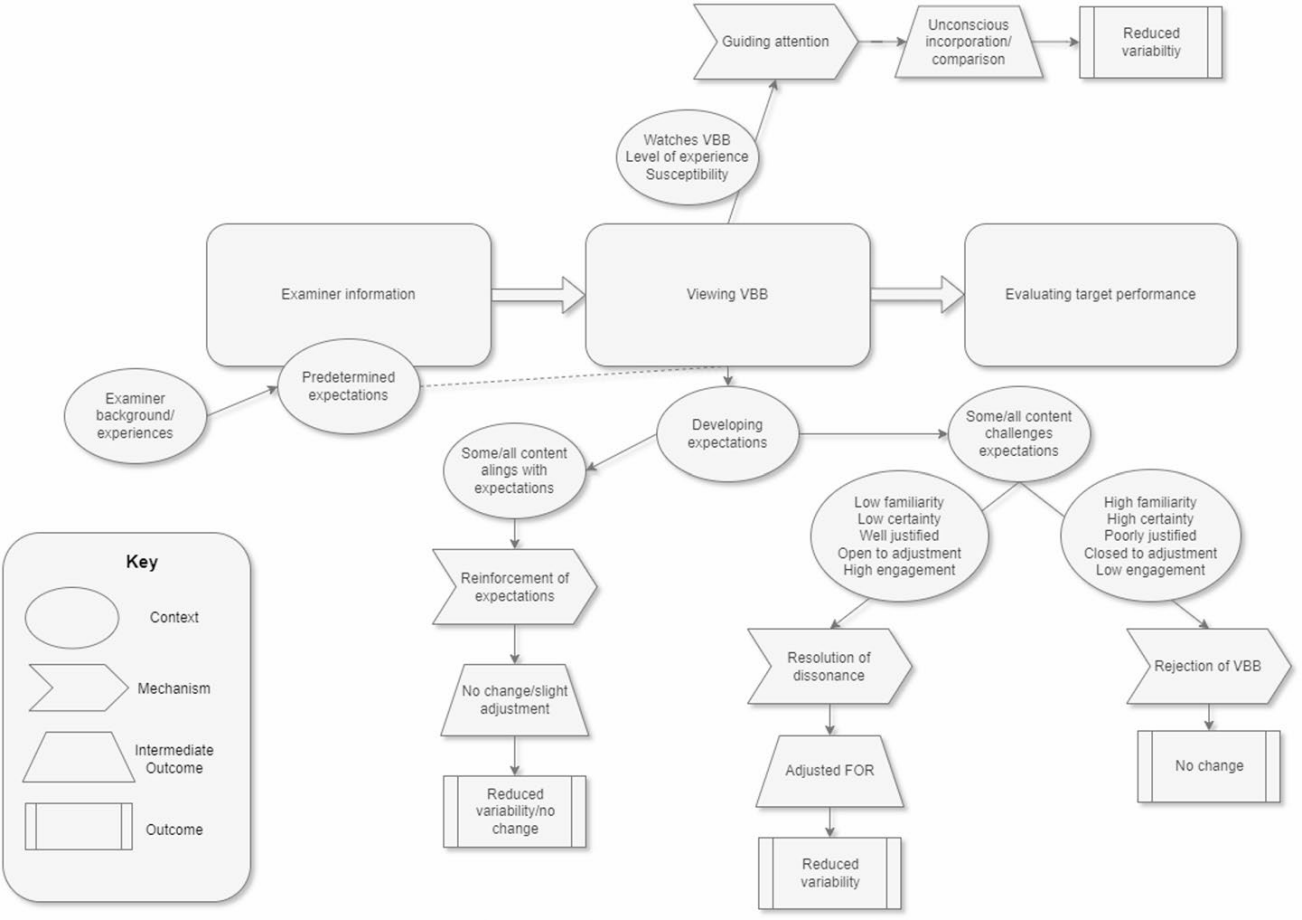
Schematic of initial programme theory

Realist Evaluation methods rely on an initial programme theory (IPT) that represents the researchers’ presumptive theory about how they anticipate the phenomenon may work, which is then refined through analysis of collected data. The IPT was developed by the authors, based on literature review, their existing understanding of assessors’ cognition, and extensive experience of OSCE examining and examiner training. The IPT is illustrated in Fig. 1.

### Methodological strategy

We gathered data through online stimulated Realist interviews (Manzano, 2016) in which participants experienced Video-based Benchmarking (i.e. the intervention) under one of a number of manipulated contexts and then discussed their experiences of and responses to the intervention.

### Population, sampling and recruitment

The study population was OSCE examiners who examine clinical years students at Keele University School of Medicine. These were purposively sampled to include a mixture of experienced and inexperienced OSCE examiners from a range of clinical backgrounds. Participants were recruited by emailing adverts to the entire database of OSCE examiners. Interested individuals were sent information about the study and completed an online consent form prior to participating.

### Intervention

The benchmarking intervention featured an OSCE station taken from a previous year 3 (first clinical year) OSCE exam at Keele University School of Medicine. The station required candidates to take a history from a returning traveller (played by a simulated patient) and then suggest appropriate differential diagnoses and investigations. The station was scored using the Generic Consultation Skills (GeCoS) domain-based marking scheme (Lefroy et al., 2011; Lefroy, 2024) which allocates a score of 1 (must improve) to 4 (very good) for 5 domains which are selected based on station content. These are added to a global score out of 7, to give a total mark out of 27.

The videos were of real student OSCE performances from prior research (Yeates et al., 2021) and participants had provided consent for their use in future research. Written examiner information for the station described the simulated patient’s history, the marking format and some guidance on how to attribute marks. Three videos from this station were used within the study. Benchmark information was developed by the research team, which included: an agreed overall score for the benchmark performance (which also indicated its category, i.e. fail, borderline, pass, good etc.) and an explanation of the reasoning for the agreed score. To generate these agreed scores and explanations, three of the study’s authors (PY, JL, RM) independently watched, scored and made detailed notes on the three performances, considered the original (live) examiner’s scores for the performances, and then met to discuss their views, and reach an agreed position on each performance. The initial panel judgements varied from 14 to 17 out of a maximum of 27 for the borderline benchmark, resolved through discussion to 15; and 20–23 out of a maximum of 27 for the good benchmark, resolved to 22. The station had a cut score of 16, meaning that the borderline stations was a minimal fail. Cut scores were assigned using the borderline regression method (Downing et al., 2006) subsequent to the OSCE in which the videos were filmed. Owing to small participant numbers this was not re-calculated or used within this study.

Participating examiners were sent the station information in advance of the interview and asked to use it to prepare to examine the station in their usual manner. The intervention was performed within the context of a realist interview, with segments of the interview conducted before, mid-way, and after the benchmarking intervention. The benchmarking procedure involved examiners watching one of two possible benchmark videos (either “borderline” or “good” performances). They were provided with benchmarking information (agreed scores and justifications) for the video either before or after observation of the video. These contexts (level of performance and timing of benchmark information) were manipulated aiming to create balanced exposure of different participants to these contexts of novice, intermediate or extensive prior OSCE examining experience. After observing and scoring the benchmark video, examiners then scored a final target video of a performance, whilst being asked to imagine that this performance was the first student in the OSCE.

### Data collection

**Fig. 2:**
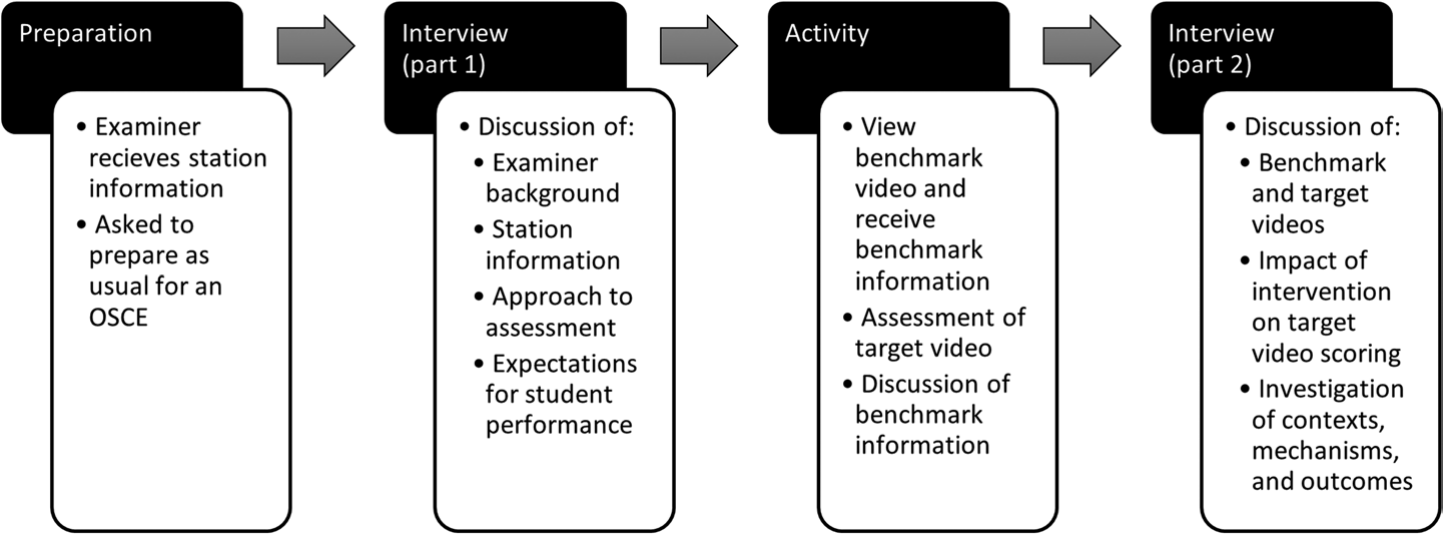
Schematic of data collection process

Figure 2 illustrates the general data collection process. Examiners provided their gender, prior examiner and assessment experience, and clinical and educational background via an online survey. Examiners’ scores for the benchmark and target performances were collected via a separate online survey during the interview. All remaining data were gathered through semi-structured interviews conducted and recorded through Google Meet. These comprised 3 parts: prior to the benchmarking procedure, interviews focused on participants’ preparation to examine the station and their baseline expectations; midway through the interview (i.e. after scoring the benchmark video but before scoring the target video), participants briefly described their reactions to the benchmarking information; after scoring the target video, interviews explored participants perceptions of the intervention and its influence on their judgement.

Interviews were conducted by RE using a realist approach. Participants were asked to describe their perceptions of preparing for and conducting OSCE exams and their impressions of the benchmarking intervention. Follow up questions probed specific theoretical ideas, testing concepts and elaborating new ideas. Questioning was guided by a topic guide based on the initial programme theory, but the questioning approach evolved in light of theory as it was developed. RE sought to establish a relaxed, non-judgemental environment, and explored emergent issues raised by participants. Initial interviews were transcribed by RE. The remainder were transcribed by an external transcription company.

### Analysis

Data were analysed according to a realist logic of analysis (Pawson & Tilley, 1997; Wong et al., 2012). NVivo 11 software (QSR, 2014) was used to organise coding. RE coded all transcripts whilst all other authors second coded at least one transcript to sensitise and broaden interpretation. Coding involved line by line reading of transcripts searching for information which was relevant to the evolving programme theory. Data was initially coded by labelling concepts and then categorizing relevant concepts together. This was done both inductively and deductively, based on the concepts in the IPT. Next, portions of relevant data were coded as either ‘Context’, ‘Mechanism’ or ‘Outcome’. As analysis progressed, different configurations of context, mechanism and outcome were trialled and refined based on demi-regularities within the evolving data (semi-predictable patterns or pathways of program functioning (Jagosh et al., 2015). This process involved review of the concept labels and the underlying text and involved the Realist principle of retroduction (identification of hidden causal forces that lie behind identified patterns or changes in those patterns (Greenhalgh et al., 2017). As context, mechanism, and outcome configurations (CMOCs) were discerned within the data, these were organised to refine and develop the initial programme theory. As the coding process was ongoing, other research team members (PY, JL and RM) read portions of data and the evolving CMOCs to bring critical challenge and refinement. This process proceeded until the researchers judged that sufficient information power (Varpio et al., 2017) had been achieved, and the updated programme theory was finalized.

### Reflexivity

The authors comprise one psychologist who had no experience of OSCEs prior to the study (RE) and three clinical academics (PY, JL, RM) who all had an interest in assessors’ cognition and experience of OSCE conduct. This research comprises the first study within RE’s PhD thesis. All authors considered the intervention a plausible potential means to enhance examiner standardisation but had no strong conviction as to its effectiveness, nor any strong conviction regarding mechanisms through which it might achieve effectiveness nor contexts which would evoke them. None of the research team use or are responsible for administering video-based benchmarking as part of their academic roles.

### Ethics

All participation was voluntary. Participants provided consent and had the right to withdraw. Ethical approval for the study was granted by Keele University Research Ethics Committee (ref MH-200111).

## Results

16 examiners participated, of whom 6 considered themselves experienced, 5 novice and 5 of intermediate experience. At least one of each group experienced each of the permutations of VBB (benchmarking information provided a/before or b/after watching either a 1/borderline or 2/good benchmark performance). Participant demographic data are shown in Table 1.

**Table 1:** Participant demographics

Variable		Count
Examiner experience of examining OSCEs (Duration in Years)	0–2 years	6
	3–5 years	5
	6–9 years	2
	10 + years	3
Clinical background	Primary care	3
	Surgery	5
	Diagnostics	1
	Child Health	1
	Emergency Medicine	1
	Obstetrics & Gynaecology	1
	Medicine	3
	Unknown	1

Analysis produced a total of 13 CMOCS which collectively comprise the knowledge claims of our refined programme theory. These are displayed in Table 2 and can be found with further supporting quotes in appendix 1. Here we present a narrative which describes the integration of these CMOCs along with selected supporting quotations.

**Table 2:** Context-Mechanism-Outcome statements

CMOC label	Statement
CMOC 1: Preparation	When conscientious examiners (C) study written examiner information prior to the OSCE (C) and engage in preparation rituals (C) they still remain uncertain about precisely what to expect from students (O) because their understanding remains abstract until they gain experience of using it to make judgements on student performances (M).
CMOC 2: Locating Judgement	When examiners encounter a concrete example of performance on the station for which they have prepared (C), along with benchmark information for the performance (C), they develop a sense of their degree of alignment with the benchmark (O) by comparing their judgement with the scores and explanations from the expert panel (M).
CMOC 3: Dissonance	When examiner’s judgement and the expert panel scores/justification substantially differ (C), examiners seek to resolve the discrepancy by either adjusting their perspective or dismissing the benchmark (O) because they experience an uncomfortable sense of dissonance in response to the discrepancy (M).
CMOC 4a: Accept	When examiners’ are dispositionally amenable to change (C), or the benchmark panel is credible (C) and the explanation is convincing (C), or they can resolve misunderstandings through discussion (C) and they have sufficient cognitive engagement with the benchmark information (C), they are more likely to align their judgements to the benchmark (O) because the benchmarking process persuades them to adopt a new perspective (M).
CMOC 4b: Dismiss	When examiners’ are dispositionally resistant to change (C), or they believe that the benchmark panel lacks credibility (C) or the benchmark information isn’t convincing (C), they are unable to resolve areas of misunderstanding through discussion (C) or they lack sufficient cognitive engagement with the benchmark information (C), they are likely to dismiss the benchmark information (O) because the benchmarking process fails to convince them to alter their perspective (M).
CMOC 5: Engagement	Examiners’ degree of cognitive engagement with the benchmark material (C) both *during observation* (C) and *subsequent reflection* on comparisons with their own scoring (C) appears critical to the effectiveness of benchmarking (O) because this enables them to integrate their reflections and make adjustments to their pre-existing schema (M).
CMOC 6: Agreed scores after video	When examiners are asked to score benchmark videos before being presented with benchmarking scores and explanations (C), they are more likely to actively engage with the videos content (O), because they are aware of a need to reach a justifiable judgement on the video (M).
CMOC 7: Agreed scores before video	When examiners are presented with benchmarking scores and explanations before watching the benchmark video (C), they are more likely to passively engage with its content (O) because they already have the assurance of knowing the expected level of the performance (M).
CMOC 8: More videos	With increasing numbers of benchmark videos (C), showing varying levels of performance (C) and more detailed explanations of the rationale for the benchmark scores (C), examiners develop a clearer sense of the expected level of performance (O) because comparing performances helps to illustrate different points on the assessment scale (M).
CMOC 9: Overload	With progressive increases in the number of videos which examiners are asked to watch (C) or increasing complexity of the explanations provided to examiners (C) examiners engagement with observing videos is reduced (O) because they feel overwhelmed by the volume of information they have to process (M) and the time requirements involved in video observation produce a pragmatic challenge to completion (M).
CMOC 10a: Timing	When examiners engage with benchmarking processes shortly before the OSCE (C), the benchmarking information is more cognitively available within their internal representation of the case (O) because they are still able to recall the performance (M).
CMOC 10b: Timing	When examiners engage with benchmarking processes immediately before the OSCE (C), the degree of assimilation of the benchmarking information into their marking schema is reduced (O) because there is insufficient opportunity to reflect on the benchmark information (M).
CMOC 11: Subsequent judgement	When receptive examiners (C) have thoroughly engaged (C) with well-constructed benchmark examples and information (C), their judgements on subsequent target performances (i.e. real students in the OSCE) are more aligned to the expected standard (O) because they can consciously (and possibly unconsciously) compare target performances to the benchmark content (M) and their internal frame of reference has adapted in response to the benchmark information (M).

### Preparing for the OSCE

Participants described preparing for OSCEs by spending time considering the written examiner information (SP script, marking rubric, candidate and examiner instructions) provided to them in advance of the OSCE by the assessments team. This required a conscientious attitude, so examiners may not always show this degree of preparation. Participants described integrating the station information with their own clinical judgement of the station to reach an initial individual interpretation of the station, including an expectation of how students would perform. This sometimes involved developing their own tools to focus or guide their observations, for example, by creating their own supplementary checklist to help to focus their attention during the exam.

*“I usually spend the evening before the OSCE going back through all… material. You know… creating my own (…) score sheets because I… I prefer to tick something off.” Examiner 2 (Procedure: after; Borderline)*^1^

As a result, participants had a baseline expectation of what to expect from students in the station which they brought to the benchmarking process. These appeared to differ between examiners, in part due to difference in clinical expertise, familiarity with the OSCE content and differences in examiners preparation for the OSCE. Despite their preparation, participants’ baseline expectations remained uncertain because they were derived from an abstract description of performance which requires experience of judging student performances on the station to clarify its interpretation [CMOC 1: Preparation].

*“Um*,* a bit worried*,* um*,* for the students [I: Yeah]. To make sure*,* um*,* I don’t*,* umm…*. *Especially the first ones you know you’re trying to, uh, be consistent and you don’t know from the first one whether you’re*,* what is… the standard you’re expecting.” Examiner 9 (Procedure: before; Borderline)*

### Locating and resolving differences

Observing and scoring a video-based benchmark performance gave participants a concrete example of performance on the station for which they had prepared. The written benchmark information for the video performance (i.e. the agreed score and justifications for that score) enabled participants to compare their judgement with that of the expert panel and to reflect on the reasons provided for the expert judgement. This let them identify their potential scoring differences, and develop a sense of their degree of alignment with the expert panel [CMOC 2: Locating Judgement]. This sense of alignment led to different reactions by participants. When the participants’ personal judgements were very close to that of the panel, they felt reassured as it indicated their judgement was aligned. Conversely, most participants felt uncomfortable when participant and expert panel judgements were discrepant because it may have implied they had erred [CMOC 3: Dissonance]. This produced cognitive dissonance (psychological discomfort emanating from conflicting beliefs or observations), which they sought to resolve:

*“Well, so if the scores had been significantly different, there would be multiple questions won’t there. Had you sent me the right video? Was I assessing the appropriate interaction? Was my understanding of the topic so far off of kilter that it was a failing on my part? Was the examiner at that time, again, was there any extrinsic factors or human factors that had meant he or she was being unduly or unfairly harsh?” Examiner 15 (Procedure: before; Good)*

*“I suppose for me, because I was pretty close to what they said, then that’s quite reassuring. Then I think, ‘Oh yeah, that’s good’ because I’m about right. If I was very different, maybe it would be less helpful, or maybe that’s more challenging to deal with then.” Examiner 4 (Procedure: before; Good)*

Participants sought to attribute the discrepancy in judgement to either an internal (they were in error), external (some other factors were responsible) locus, or a combination of both (they were in error because something external threw them off their usual approach, perhaps therefore not warranting adjustment to their internal frame of reference). Depending on this attribution, participants then tried to adjust their perspective to align with the expert panel, or, conversely, dismissed the benchmark information and retained their existing position [CMOC 3: Dissonance]. To align with the expert panel and attempt to alter their internal representation of the judgement task, participants needed to be persuaded by the information presented to them. Several contexts either favoured or hindered alignment by participants [CMOC 4a: Accept]. Some participants were amenable to change (which may have related to their degree of certainty in the station), whilst others were more adamant that their judgement was correct perhaps because the station focused on a clinical context which was central to their scope of practice. The credibility of the benchmark panel (i.e. composed of individuals with the requisite expertise to judge) and the explanatory clarity of the justifications (participants could logically see how the justification related to the performance and the marking scheme) were both critical to the credibility of the benchmark. Alignment was promoted by participants having sufficient cognitive resources to process the information (i.e. enough time, not multi-tasking) and the opportunity to discuss any misunderstandings with other participants. Conversely, if any of these contexts were absent, then the likelihood of participants dismissing the benchmarking information and retaining their original perspective was increased [CMOC4b: Dismiss].

### Modulating engagement

Participant engagement with benchmarking appeared critical. Engagement was judged by the interviewer based on participant observation and so is difficult to evidence through quotes. In some cases, participants appeared distracted, e.g. checking their phone; talking to others in the room, which was considered to reflect passive engagement. On the other hand, some participants appeared to watch the videos intently, others voluntarily scored the benchmark video or took notes, which was interpreted as active engagement. Participants’ cognitive engagement both *during* observation of videos and *subsequent reflection* on comparisons with their own scoring, was critical as it appeared to mediate participants’ ability to integrate their observations and reflections on the benchmarking information in order to make adjustments to their pre-existing schema [CMOC 5: Engagement].

A number of procedural contexts seemed to facilitate mechanisms which increased participants’ engagement. When participants scored the benchmark performances without being aware of the expert judgement (i.e. using the “information after” procedure), they were aware of a need to reach a justifiable judgement on their own accord and their engagement increased [CMOC 6: Agreed scores after video]. Conversely, participants felt that knowing the expert panel scores and justification before observing the video (i.e. the “information before” condition) reduced this pressure, giving a sense of reassurance which altered how they watched the video, thereby reducing engagement [CMOC 7: Agreed scores before video]. Examiners across conditions felt that receiving the information after the performance example facilitated independent scoring and clarity in comparison to the provided information, and created a learning experience they could use in the following assessment. Conversely, examiners receiving information before the example, who tended to engage more passively, found more of a challenge in learning from the experience.

*“I think it might… almost change the way you watch the performance (…) ‘cause you might go, ‘Oh, Yeah yeah yeah, no. I absolutely agree’ when you haven’t really thought about it [referring to receiving the benchmark information before viewing the video] in any sort of great detail, whereas if you’re scoring it yourself, you’ve you’ve had to sort of think about it.” Examiner 2 (Procedure: after; Borderline)*

While no overall differences were observed between novice and experienced examiners in how they experienced the intervention, novice examiners tended to comment on the usefulness of VBB in relation to their (comparatively limited) experience, and theorised that more experienced examiners may be less likely to engage with VBB. However, as all examiners in the cohort commented positively on the intervention, this observation is based on participant supposition rather than direct experience. As a result, we can’t make a strong assertion that the intervention was more effective for novice examiners, so we have not included it in the final theory. Nonetheless, we include this evidence to enable readers to appraise this perspective:

*“Somebody like me that’s only just starting in OSCE examining probably would really appreciate benchmarking a lot more*,* perhaps*,* than somebody who feels that they’re very comfortable with being an OSCE examiner and don’t want to spend the extra time doing benchmarking.” Examiner 16 (Procedure: after; Borderline)*

Although these variables were not manipulated in this study, participants believed that multiple benchmark videos showing different levels of student performance (i.e. borderline vs. good) or more detailed explanations of the expert panel’s rationale would illustrate the different points on the assessment scale through comparison, thereby helping participants to develop a clearer sense of the expected level and range of performance [CMOC 8: More videos]. This desire was present for more novice examiners, perhaps because their comparative lack of experience produced decreased levels of confidence in their examiner ability:

*“Because it’s easy to tell when someone has got, kind of, unsatisfactory. I think the borderline ones are the difficult ones, so maybe having like, someone who’s definitely passed, and then someone who’s more borderline, and then watching the two and having a go at benchmarking the two. That would be really useful, yeah.” Examiner 3 (Procedure: before; Borderline)*

*“I think it would be nice to see both a less competent and the more competent one so that you can see the top and the bottom of the ranges. I think I would prefer that. (…) That might be because of my own lack of confidence in OSCEs because I’m limited in my experience of them. But*,* yes*,* the more the merrier.” Examiner 16 (Procedure: After; Borderline)*.

An increase in benchmarking information may increase the effectiveness of the intervention but would require greater engagement and may become overwhelming, thereby reducing engagement [CMOC 9: Overload].

*“I think watching more at different levels would have some… I suppose it would have some benefit. But I think possibly, it would be a somewhat diminishing return and each extra one that you watch would be a further time constraint as well.” Examiner 11 (Procedure: before; Borderline)*

Participants judged that two to three videos, depending on length, seemed an optimal compromise between comparative information and the load placed on participants. Similarly, participants felt that benchmarking should occur shortly before the OSCE to ensure that the information was cognitively available [CMOC 10a: Timing]. They recognised that OSCE days are pressured and that performing the benchmarking process immediately before the OSCE (i.e. within the hour preceding it) may limit the time and cognitive resources to reflect on the benchmark and therefore its assimilation [CMOC 10b: Timing]. They judged that approximately 24 h before the OSCE may be the optimal timing for benchmarking:

*“I think that discrepancy might be quite hard to reflect and act on just before starting examining in the morning. So*,* I think yeah*,* maybe the day before it would be fresh in your mind*,* but it would still give you a bit of time extra to have another read through the mark scheme and have another chat with the other examiners.” Examiner 3 (Procedure: before; Borderline)*

#### Influence on subsequent judgements

After the benchmarking intervention, participants then judged a target performance, which was intended to replicate the experience of judging the first “live” student in the OSCE. This gave participants an opportunity to describe their perception of the impact of the benchmarking intervention on that judgement. This varied based on participants’ receptivity to and engagement with the benchmarking intervention. When receptive participants thoroughly engaged with well-constructed benchmark information, they perceived that their judgements on the subsequent student performance (i.e. the proxy for live students in the OSCE) was more aligned with the expected standard. The opportunity to compare and reflect on their scoring of the benchmark video in relation to the expert panel scores and justifications enabled them to adjust their internal frame of reference. They perceived that they were then able to use this adjusted frame of reference to judge the subsequent (‘target’) performance in a manner which more closely aligned with expectations [CMOC 11: Subsequent judgement].

*“It was like trying to set my own standard between the two, because you do compare as to what you said in the first video [i.e. the benchmark video] in terms of the feedback, in terms of the reasoning, my reasoning for marking and scoring, and then to use the same standard, the same level of expectation from the candidate.” Examiner 7 (Procedure: after; Good)*

When the benchmarking intervention operated positively in this way, participants perceived its effect (or outcome) on their subsequent judgements in a number of different ways. Firstly, several described a warm-up effect over the first few candidates in the OSCE during which they gain confidence and ‘get their eye in’, a process whereby they experientially gain familiarity with the station over a few candidates: when the benchmarking intervention was effective, it reduced the need for them to ‘get their eye in’, making them more confident and decisive with initial students. They also perceived that benchmarking would align examiners both with the intended standard of the exam (i.e. resulting in more accurate categorisation of student performances) and would also help to align examiners in different parallel circuits of the OSCE, thereby aiding standardisation of judgements across parallel circuits.

## Discussion

### Summary of middle-range theory

Video-based benchmarking supported examiners’ OSCE preparation. Despite conscientious preparation, examiners are often uncertain about what to expect from candidates and how to score. Observing and judging concrete examples of performance enabled examiners to compare their judgement with that of the expert panel and enabled them to locate their initial judgements in relation to the agreed standard. Whilst this reassured examiners who were closely aligned, it produced dissonance for examiners who were not. Examiners resolved this dissonance by effortfully aligning their frame of reference with the expert panel or dismissing it. Contexts which favoured such conscious realignment by examiners (via a variety of mechanisms) included amenability to change, lower certainty in their judgement, panel credibility, informational clarity, ability to discuss information and the degree to which examiners engaged with observation and reflection. Multiple contexts influenced the extent of examiner engagement through a variety of mechanisms, including conscientiousness and the timing of benchmark information in relation to videos. While more videos (at different levels of performance) will increase the calibrating information available to examiners, this may reduce engagement through overload. Sufficient time to reflect on performances will further enhance engagement. When conscientious examiners engaged with well-constructed benchmark information, they perceived that their judgements became more aligned with the expert panel, improving their scoring of subsequent candidates.

### Relationship of findings to existing theory

The observation that examiners felt uncertain in the judgements they were about to make despite conscientious preparation accords with prior studies from both workplace-based assessments and OSCEs (Rothman et al., 1996; Yeates et al., 2013a). This suggests that examiners may find it difficult to cognitively operationalise written descriptions or rubrics. Whilst not claiming that VBB will completely remove this uncertainty, it appeared within this study to increase confidence. This suggests that examiners may find it easier to understand a judgment task through comparison with exemplars than through abstract descriptions of expected performance. This accords with theories of exemplar-based reasoning in human judgement (Bröder et al., 2017), which posit that humans categorise objects by similarity with examples stored in memory rather than by reasoning from typical features. As a result, the availability of relevant examples would be expected to make judgement easier (Manis et al., 1993).

Examiners’ experience of cognitive dissonance in relation to discrepancies with the expert panel is also of interest. Cognitive dissonance is a negative affective state that results from an individual experiencing two discrepant cognitions (Cooper, 2012). An extension to this theory, known as the self-consistency model, suggests that individuals experience dissonance only if their sense of self is part of the cognitive discrepancy they experience (Hinojosa et al., 2017). Given this, we may deduce that for at least some examiners, their beliefs about performance as an examiner are very strongly held and personally important. This accords with prior research which has described examiners feeling discomfort when they realise that they are outliers relative to their peers (Crossley et al., 2019). Given this it is not surprising that at least some examiners will try to find ways to dismiss benchmark information by challenging its credibility: challenging external sources is a well-recognised self-preservation mechanism (i.e. ‘my judgement is still correct’) to resolve cognitive dissonance (Capraro, 2024).

Some of our findings relating to the timing of benchmark information also hint at the potential operation of defined cognitive biases. The difficulty that examiners experienced in reaching an independent judgment if the expert panel score was made available to them before watching the performance may be a case of “anchoring” in which a person finds it hard to move away from an already suggested value (Furnham & Boo, 2011; Tversky & Kahneman, 1974). Equally, in this scenario examiners may have accorded with the panel score due to the “Bandwagon effect”, a form of conformity in which a person goes along with the judgement of a crowd in order to fit in rather than reaching an independent judgement (Bindra et al., 2022). When examiners are only presented with the expert panel score after observing the performance, there is still the potential for bias if they do not have to commit to a score for the performance. In this scenario, they might be expected to experience hindsight bias (Calvillo, 2012) in which someone unduly believes that they would have reached the same judgement as the one they are told is correct. As all of these effects would reduce the examiner’s tendency to reach a judgement which genuinely reflects their own internal frame of reference, they would all be expected to reduce the utility of the comparison of the examiner’s judgement with the benchmark and, therefore, the effectiveness of the intervention. For this reason, it seems that VBB is most likely to be effective if examiners score performances and then subsequently receive benchmark information.

As described in the background, the intent of our study relies on the assumption that assessor variability is “error”. This contrasts the legitimate difference interpretation of assessor variability which is espoused by social constructivist views of assessment (Gingerich et al., 2014). These assert that different perspectives of a learner’s performance offer greater information about the range of ways their performance may be interpreted and therefore broader feedback and greater opportunity to improve. From this perspective, aligning assessors would *reduce* the information provided to learners and is therefore undesirable. Recent research in this domain has shown assessors using multiple adaptive frames of reference within a holistic longitudinal judgement process which is used to foster independent practice (de Vos et al., 2024). Tavares et al. have argued that rather than resolving these different perspectives, it matters more to align assumptions about error with the purpose of the assessment (Tavares et al., 2019). Given that in many uses of OSCEs, which are treated as a single-point in time assessment to determine progression, students’ scores become the overarching concern. In this scenario, treating examiner variability as error is consistent with the assessments purpose and VBB may be beneficial. Notably, though, in constructivist-orientated programmes of assessment (such as CBME) (Gingerich et al., 2014) where the aim is to maximise longitudinal growth through triangulating feedback and coaching, VBB may not align with the assessment’s purpose and so could (at least in theory) be detrimental.

### Recommendations for practice

Whilst it is premature to make firm recommendations for practice from a single study, we offer tentative suggestions which may promote successful use of benchmarking in practice. These include presenting examiners with two or three videos of performances (depending on their duration) depicting varied levels of performance (perhaps good and borderline), and (following sufficient engagement with station information) asking examiners to score each performance prior to receiving the panel score and justification for the performance. The benchmark panel must be credible (i.e. sufficiently and currently clinically experienced in the question domain) and the explanations must be clear and relate clearly to both the marking scheme and performance. Examiners may benefit from discussing the benchmark information with other examiners. Benchmarking should be within 24 h of the OSCE, but perhaps not immediately before, as this may limit the time for reflection. Further, as it is recognized that sufficient sampling across examiners and stations is the most effective method to address examiner variability (Eva, 2018), and given the likely resource implications of implementing VBB, if it is not realistic to use VBB across all stations and examiners then institutions may choose to use VBB strategically, focusing its use on either assessment contexts or groups of examiners where variability is notably problematic.

### Limitations

We assert that we have conducted a rigorous study which offers considerable novel insight into this phenomenon. Despite this, all research has limitations. Our study was limited by the context in which it was conducted: a single institution and a single OSCE station. In particular, with the present study using only a history taking station, there may be limitations in the application of this intervention to other station types. Rates of assessor agreement can vary between stations of different topics within the same specialty (Touma et al., 2023), and there may be differences in examiner reliability between communication skills and clinical skills (Brannick et al., 2011). Furthermore, there may be differences in examiners’ preconceived expectations that may impact if and how VBB may work, as different station types assess different skillsets. As a result, the results may not implicitly generalise to other contexts, and therefore research into other station types is needed. All of our participants were necessarily volunteers, who may therefore have been more motivated to engage than is representative of general examiners. This could be considered in future work examining the use of VBB in practice. Our study has focused on the cognitive aspects of examiners’ judgements, but we have not examined social or sociological contexts or how these may produce additional mechanisms which could further influence the outcomes of benchmarking. Furthermore, we were unable to look at the impact on scoring due to the small sample, or quality of feedback information, as views of performances were explored within the interviews which differs to the way feedback is usually provided by assessors within the exam context. These should be explored in further research. Lastly, we have reported examiners’ perceptions of the influence of benchmarking on their subsequent judgement. As perceptions of judgements and the actual judgements which people may make can differ (Nisbett & Wilson, 1977), we are not able to say whether benchmarking actually does produce greater alignment of examiners’ judgements through the contexts and mechanisms we have suggested. This will require demonstration through more objective methods such as randomised experiments.

### Suggestions for future research

To extend this initial insight into the operation and use of video-based benchmarking in OSCEs, future research should explore these issues across additional contexts (a range of OSCE stations, different institutions, and levels of learner) and explore sociological as well as cognitive influences on judgements. Experimental research should determine the influence of video-based benchmarking on examiners’ score alignment.

### Key practice findings

Given our findings, and whilst acknowledging the need for further research prior to making any firm recommendation, we tentatively suggest that if VBB is to be used in practice the following design features of the VBB procedures may increase the likelihood of them enhancing examiner alignment:

- The benchmark performance content should be specific to the target OSCE to be assessed by the examiner.
- VBB should take place near-in-time to the target OSCE (within 24–48 h).
- The benchmark information should be presented to examiners after viewing the relevant example performance.
- The benchmark information should be convincing, clear and from a credible source.
- Examiners may benefit from the opportunity to discuss the content of VBB.

## Conclusions

Video-based benchmarking is a complex intervention which has the potential to enhance examiner alignment in OSCEs but operates differently for different people under different circumstances. Whilst noting the need for further research we tentatively suggest a number of contexts which may make VBB more likely to achieve beneficial outcomes and the mechanisms through which these effects are achieved.

## Electronic supplementary material

Below is the link to the electronic supplementary material.

Supplementary Material 1

## Data Availability

Data is provided within the manuscript or supplementary information files.

## Footnotes

1 The notation ‘Examiner 2 (Procedure: after; Borderline)’ indicates that Examiner 2 watched the benchmarking video which showed borderline performance and received the written benchmark information after they finished observing the video. Similarly, ‘Examiner 4 (Procedure: Before; Good)’ indicates that examiner 4 watched the benchmarking video which showed good performance and received the written benchmark information before they began observing the video
