# Supplementary material for: Understanding contexts and mechanisms through which video based benchmarking promotes alignment of examiners’ scoring in objective structured clinical exams
Source: Adv Health Sci Educ Theory Pract. 2025 Jul 4;31(2):473–94. doi: 10.1007/s10459-025-10454-3 (PMC13046630; doi:10.1007/s10459-025-10454-3)
Supplement: Supplementary file 1 — Supplementary Material 1 [file 10459_2025_10454_MOESM1_ESM.docx]

## Appendix 1: CMOCs with supporting text

Appendix 1 shows the context-mechanism-outcome configurations (CMOCs) underpinning the resulting programme theory that we developed through the research process. A summary of each CMOC is provided, followed by the supporting data. Within each CMOc, we have indicated contexts with (C), mechanisms with (M), and outcomes with (O).

**Key:**
P = Participant

I = Interviewer

When no speaker is identified, the participant is speaking.

Sources are labelled in the following format: Examiner 2 (Procedure: After; Borderline)
This indicates the source is participant 2, who followed the procedure permutation where they received the benchmark information after viewing the benchmark video, and they viewed the borderline benchmark performance.

**CMOC1: Preparation**

When conscientious examiners (C) study written examiner information prior to the OSCE (C) and engage in preparation rituals (C) they still remain uncertain about precisely what to expect from students (O) because their understanding remains abstract until they gain experience of using it to make judgements on student performances (M).

“I usually spend the evening before the OSCE going back through all the sort of material. You know... creating my own (…) score sheets because I... I prefer to tick something off rather than to to scribble 'cause I can't read my own handwriting sort of half the time when I'm writing things down”
Examiner 2 (Procedure: After; Borderline)

“Um, a bit worried, um, for the students [I: Yeah]. To make sure, um, I don't, umm... Especially the first ones you know you're trying to, uh, be consistent and you don't know from the first one whether you're, what is … the standard you're expecting.”
Examiner 9 (Procedure: Before; Borderline)

“I guess because you just kind of have that sense of how important it is to get it right, because you don't want to sort of fail someone who shouldn't be sort of failing at station. But similarly, you don't want to pass someone if they're not ready to pass. So, I think there's a lot of kind of responsibility with OSCE examining, and I think it makes you a little bit apprehensive at the start, definitely.”
Examiner 3 (Procedure: Before; Borderline)

“I think it's better to actually have an idea of what to expect and the questions (I: Mhmm, mhmm] uh, and go a bit, but a bit of preparation. I mean I know everything is there and we need, we should be able to manage with what is given in the the sheet, but, umm, yeah, it it helps to have a bit of a background, um uh beforehand, yes.”
Examiner 9 (Procedure: Before; Borderline)

“And also, on the day of the OSCE, if we’re doing it in two circuits, to turn around to the chap or chap-ess, whoever is doing it on the other circuit, and say, look, how are you going to judge this and how are you going to assess this particular OSCE?”
Examiner 13 (Procedure: After; Borderline)

“as long as you've got kind of a thorough mark scheme, lots of detail, and I guess it helps to have kind of the huddle with the other examiners on the circuit at the start as well. And hopefully one of those would be the specialist, and maybe you can then kind of say is there anything else that they might say are there any other kind of management things that they might say that's not written in here”
Examiner 3 (Procedure: Before; Borderline)

“I guess if you know that it's a benchmark video, so you can watch it, you can have a go at kind of how would I approach this if I was marking this?”
Examiner 3 (Procedure: Before; Borderline)

“I think maybe if I had watched the benchmark video and then you gave me the scores, I guess it's, that's similar to how we do the OSCE examiner training. And I think that is affective because I could have a go marking it myself”
Examiner 3 (Procedure: Before; Borderline)

“I think it’s quite useful. I mean, again time and resource are always going to be the biggest issues aren’t they [I Yeah, yeah] in terms of being able to say, well actually have you actually got the time to be able to prep people, and… almost, kind of, for an examiner to be able to get their eye in before they go and they’re actually exposed to student. So if you can almost have that beforehand, that’s great. Because then you’re not worried about what’s happened to the first 2 or 3 students that they’re seeing. Because actually, they’ve already, kind of got their reference point in, and they know what they’re looking for and where the mark scheme should be.”
Examiner 5 (Procedure: Before; Borderline)

**CMOC2: Locating judgement:**

When examiners encounter a concrete example of performance on the station for which they have prepared (C), along with benchmark information for the performance (C), they develop a sense of their degree of alignment with the benchmark (O) by comparing their judgement with the scores and explanations from the expert panel (M).

“If you're scoring it yourself, you've had to sort (…) think about your sort of justifications, and then compare that with somebody else’s justifications and then that gives you an idea about whether… You're being a little too harsh, too easy, you know, whether you see things completely different”
Examiner 2 (Procedure: After; Borderline)

“Yeah, I think it would have been helpful… just, again, to have kind of, have a go and then I guess get some feedback in terms of where your marking is, in regards to this panel of examiners who have already benchmarked it. And then you can see whether you're marking is maybe too harsh or too lenient. And then you can maybe pitch the future marking depending on that, when you get a bit of feedback.”
Examiner 3 (Procedure: Before; Borderline)

**CMOC 3: Dissonance**

When examiner’s judgement and the expert panel scores/justification substantially differ (C), they seek to resolve the discrepancy by either adjusting their perspective or dismissing the benchmark (O) because they experience an uncomfortable sense of dissonance in response to the discrepancy.

“Well, so if the scores had been significantly different, there would be multiple questions won’t there. Had you sent me the right video? Was I assessing the appropriate interaction? Was my understanding of the topic so far off of kilter that it was a failing on my part? Was the examiner at that time, again, was there any extrinsic factors or human factors that had meant he or she was being unduly or unfairly harsh?”
Examiner 15 (Procedure: Before; Good)

“I suppose for me, because I was pretty close to what they said, then that’s quite reassuring. Then I think, ‘Oh yeah, that’s good’ because I’m about right. If I was very different, maybe it would be less helpful, or maybe that’s more challenging” to deal with then.”
Examiner 4 (Procedure: Before; Good)

**CMOC 4a: Accept**

When examiners’ are dispositionally amenable to change (C), or the benchmark panel is credible (C) and the explanation is convincing (C), or they can resolve misunderstandings through discussion (C) and they have sufficient cognitive engagement with the benchmark information (C), they are more likely to align their judgements to the benchmark (O) because the benchmarking process persuades them to adopt a new perspective (M).

**CMOC 4b: Dismiss**

When examiners’ are dispositionally resistant to change(C), or they believe that the benchmark panel lacks credibility (C) or the benchmark information isn’t convincing (C), they are unable to resolve areas of misunderstanding through discussion (C) or they lack sufficient cognitive engagement with the benchmark information(C), they are likely to dismiss the benchmark information (O) because the benchmarking process fails to convince them to alter their perspective (M).

“Maybe people who have a lot of experience of doing OSCE might feel they already benchmarked themselves against themselves in the past. I don’t know. Somebody like me that’s only just starting in OSCE examining probably would really appreciate benchmarking a lot more, perhaps, than somebody who feels that they’re very comfortable with being an OSCE examiner and don’t want to spend the extra time doing benchmarking.”
Examiner 16 (Procedure: After; Borderline)

“But I think knowing that maybe like a panel of experienced examiners have seen a video and that's what they kind of pitch that benchmarking video as… I think that helps. Because then you can kind of tailor your thoughts and maybe it might make you a little bit more accurate per say for future candidate videos.”
Examiner 3 (Procedure: Before; Borderline)

“I think, to feel like there has been a group of people who have sort of sat down and considered it in some detail, and that’s about the ballpark that they think is right. I think that is quite a helpful thing.”
Examiner 4 (Procedure: Before; Good)

“In medicine, we’re taught to have lifelong learning, and I think OSCEs are the same. So, I will modify my decisions if I get good evidence to the contrary.”
Examiner 13 (Procedure: After; Borderline)

“You would want some sort of discussion to see why that is (referring to large differences between examiner scores and pre-assigned scores). I think it would be unhelpful to go into an exam and therefore mark this cohort of students, having to make a big change to that without understanding why.”
Examiner 11 (Procedure: Before; Borderline)

“I think if it was very different [referring to difference in scores] (…) that would make me think quite hard about, well why did I think so differently to this other group of people? (…) I would hope that I would be, sort of, open to thinking about their justifications, and sort of looking at mine, and, you know, adjusting mine if needed. But, I suppose if you really firmly believed differently to what they said, then that puts you in a difficult position, doesn’t it?”
Examiner 4 (Procedure: Before; Good)

“For example, in the last slot of the management, two to three, I would very openly disagree with whoever puts a two for the candidate for the management section. I don't think watching the video before or looking at the marks before would have influenced me or convinced me to say that is two. I don't think so.”
Examiner 12 (Procedure: After; Good)

**CMOC 5: Engagement**

Examiners’ degree of cognitive engagement with the benchmark material (C) both during observation (C) and subsequent reflection on comparisons with their own scoring (C)) appears critical to the effectiveness of benchmarking (O) because this enables them to integrate their reflections and make adjustments to their pre-existing schema (M).

Please note: engagement of examiners in the present study was gauged by participant observation. In some cases, participants appeared distracted, e.g. checking their phone; talking to others in the room, which was determined to be reflective of passive engagement. On the other hand, some participants appeared to watch the videos intently, and others engaged in voluntary scoring of the benchmark video or notetaking behaviour, which was interpreted as active engagement with the benchmarking process.

**CMOC 6: Agreed scores after video**

When examiners are asked to score benchmark videos before being presented with benchmarking scores and explanations (C), they are more likely to actively engage with the videos content (O), because they are aware of a need to reach a justifiable judgement on the video (M).

“I think it might... almost change the way you watch the performance (…) 'cause you might go, ‘Oh, Yeah yeah yeah, no. I absolutely agree’ when you haven't really thought about it [referring to receiving the benchmark information before viewing the video] in any sort of great detail, whereas if you're scoring it yourself, you've you've had to sort of think about it”
Examiner 2 (Procedure: After; Borderline)

“It would make me reflect more on my own performance rather than the candidate’s performance (referring to receiving the benchmarking information after viewing the video). Had I been fair and just, and did I honestly believe that I had marked unbiasedly. So I think knowing the scores retrospectively would… The scores are the scores, aren’t they? It’s what I felt was the correct at the time, but it would give me more insight into my own. Because I know I’m a dove. I know I over mark. I know I’m on the candidate’s side. So knowing the scores afterwards would just keep me on an even keep I think.”
Examiner 15 (Procedure: Before; Excellent)

**CMOC7: Agreed scores before video**

When examiners are presented with benchmarking scores and explanations before watching the benchmark video (C), they are more likely to passively engage with its content (O) because they have already have the reassurance of knowing the expected level of the performance (M).

“I So what did you think about that student’s performance?

P So, I'm just, I was kind of looking at the mark scheme as I was going through and then just jotting a few things down and trying to kind of think if you haven't told me what they had marked at, what would I have marked it as?”
Examiner 3 (Procedure: Before; Borderline)

“I think seeing it… Seeing the scores at the beginning would bias my scoring for that video. I think I would. I would like to. I think the instructions should be that what, for me at least, I would certainly like to mark it myself and then compare.”
Examiner 9 (Procedure: Before; Borderline)

**CMOC 8: More videos**

With increasing numbers of benchmark videos (C), showing varying levels of performance (C) and more detailed explanations of the rationale for the benchmark scores (C), examiners develop a clearer sense of the expected level of performance (O) because comparing performances helps to illustrate different points on the assessment scale (M).

“Because it’s easy to tell when someone has got, kind of, unsatisfactory. I think the borderline ones are the difficult ones, so maybe having like, someone who’s definitely passed, and then someone who’s more borderline, and then watching the two and having a go at benchmarking the two. That would be really useful, yeah.”
Examiner 3 (Procedure: Before; Borderline)

“I think it would be nice to see both a less competent and the more competent one so that you can see the top and the bottom of the ranges. I think I would prefer that. (…) That might be because of my own lack of confidence in OSCEs because I’m limited in my experience of them. But, yes, the more the merrier.” Examiner 16 (Procedure: After; Borderline)

However:

**CMOC 9: Overload**

With progressive increases in the number of videos which examiners are asked to watch (C) or increasing complexity of the explanations provided to examiners (C) examiners engagement with observing videos is reduced (O) because they feel overwhelmed by the volume of information they have to process (M) and the time requirements involved in video observation produce pragmatic challenge to completion (M).

“I think watching more at different levels would have some… I suppose it would have some benefit. But I think possibly, it would be a somewhat diminishing return and each extra one that you watch would be a further time constraint as well.”
Examiner 11 (Procedure: Before; Borderline)

“You know, there is a downside to… OSCE examining, particularly, you know, if you are doing the benchmarking on the day, is a long day anyway to do to do the examining. And then, if you add in a lot of other stuff beforehand (…) do you just end up losing examiners because they, it’s all just a bit too much. (…)(…) I guess, I would imagine that if you watch more than one video then it’s a more robust process, but.. but I guess, you know, you need to look, yeah, you need to do the research to find out whether that is true, wouldn’t you?”
Examiner 4 (Procedure: Before; Good)

**Timing of Benchmark**

**CMOC10a: Timing**

When examiners engage with benchmarking processes shortly before the OSCE (C), the benchmarking information is more cognitively available within their internal representation of the case (O) because they are still able to recall the performance (M).

**CMOC10b: Timing**

When examiners engage with benchmarking processes immediately before the OSCE (C), the degree of assimilation of the benchmarking information into their marking schema is reduced (O) because there is insufficient opportunity to reflect on the benchmark information (M).

“I think probably the night before would be better [referring to the best time for the intervention]. Because I think if then there's any, maybe questions or anything, that you wanted to ask about it, then it would help you consolidate what you had seen. And also give an opportunity in the morning to kind of check things or discuss things again in the huddle session with the other examiners. Make sure everyone's on the same page.”
Examiner 3 (Procedure: Before; Borderline)

“I think that discrepancy might be quite hard to reflect and act on just before starting examining in the morning. So, I think yeah, maybe the day before it would be fresh in your mind, but it would still give you a bit of time extra to have another read through the mark scheme and have another chat with the other examiners.”
Examiner 3 (Procedure: Before; Borderline)

**CMOC11: Subsequent judgement**

When receptive examiners (C) have thoroughly engaged (C) with well-constructed benchmark examples and information (C), their judgements on subsequent target performances (i.e. real students in the OSCE) are more aligned to the expected standard (O) because they can consciously (and possibly unconsciously) compare target performances to the benchmark content (M) and their internal frame of reference has adapted in response to the benchmark information.

“It was like trying to set my own standard between the two, because you do compare as to what you said in the first video [i.e. the benchmark video] in terms of the feedback, in terms of the reasoning, my reasoning for marking and scoring, and then to use the same standard, the same level of expectation from the candidate, assuming that they are both of the same training. I assumed that they were both the same... experience yeah”
Examiner 7 (Procedure: After; Good)

“She seemed to be a bit warmer in her approach and her interaction with the patient and less clinical, (…). So that was the difference between the two approaches during their history taking. Yeah, I think the first candidate [referring to benchmark video] was a bit more open in questioning and that’s why she got a bit more overall which just attests what I was saying about the second candidate [the target video]”
Examiner 7 (Procedure: After; Good)

“Well, just to reassure you that this is what you are looking for and you always would then hark back to the standardisation really or the benchmark and see just what is expected at this level from these students. And if anybody deviates from this benchmark, either for the positive or for the negative, you can then decide what marks you want to give.”
Examiner 12 (Procedure: After; Good)

“I think, you know, if I’d watched the first student and thought, yes, you know, I think that they’re probably okay but they’ve missed those bits out, I would score them as a two in that category, two in that category, a three in that category and an overall of a four. And then, to get the feedback that the overall body, the expert panel marked them slightly higher maybe, then I think it would’ve been easier to say, okay, maybe I need to be slightly more flexible with my marks on that [I: Mhmm], but perhaps more robust in my marks for the management bit.”
Examiner 11 (Before, Borderline)
